# Strongyloides Hyperinfection Syndrome (SHS) in a Patient With Prolonged Intensive Care Unit (ICU) Exposure: A Case Report

**DOI:** 10.7759/cureus.76106

**Published:** 2024-12-20

**Authors:** Julius M Nagaratnam, Alexandra Richmond, James D Perry

**Affiliations:** 1 Family Medicine, Charleston Area Medical Center, Charleston, USA; 2 Family Medicine, West Virginia University School of Medicine, Charleston, USA; 3 Pulmonary and Critical Care Medicine, West Virginia University (WVU) Medicine - Thomas Memorial Hospital, Charleston, USA

**Keywords:** gram-negative bacteremia, icu (intensive care unit), icu mortality, strongyloides, strongyloides hyperinfection syndrome

## Abstract

Strongyloides hyperinfection syndrome (SHS) is a severe manifestation of the Strongyloides parasite, often occurring in immunocompromised patients due to the inability to subdue larvae autoinfection. As the parasitic burden increases, the patient can develop worsening respiratory symptoms that mimic common pathologies such as chronic obstructive pulmonary disease (COPD). The parasite is endemic to the Appalachian region as well as subtropical and tropical areas worldwide. Despite millions of cases reported annually, it is not commonly screened for in symptomatic high-risk patients. The mortality rate from SHS is high, and few studies have explored the effects of early Strongyloides screening on reducing mortality. This case report focuses on a 71-year-old male who was diagnosed with SHS after one month of progressive deterioration in the intensive care unit (ICU) from *Escherichia coli* sepsis and acute respiratory failure. The significance of this case is to highlight the occurrence of SHS in immunocompromised patients and discuss the importance of early Strongyloides screening to mitigate mortality of symptomatic high-risk patients in endemic regions.

## Introduction

Strongyloidiasis is a parasitic infection commonly caused by the helminth *Strongyloides stercoralis* [1]. Strongyloides are endemic to many tropical and subtropical regions worldwide and are highly prevalent in the Appalachian region of the United States, consisting of states like Kentucky, Tennessee, and West Virginia [2]. Previously, the worldwide prevalence of Strongyloides was reported as 30-100 million cases annually [3]. However, a spatiotemporal statistical model approach completed in 2020 estimated the global prevalence as being 613.9 million cases annually worldwide [1,4], with an estimated 2% to 7% prevalence rate of Strongyloides in the United States [2,5].

The typical transmission route of the Strongyloides parasite is via exposed skin penetration by larvae when in contact with contaminated soil [5]. The larvae then enter the cardiovascular system and travel to the lungs, where they are expectorated and subsequently swallowed into the gastrointestinal system [5]. In the small intestines, the larvae mature into adult female worms [4] that deposit eggs in the intestinal lumen [6]. The eggs hatch into larvae, which are either expelled via the gastrointestinal system or penetrate the intestinal mucosa to infect other organs, a process known as autoinfection [6].

Most patients infected with Strongyloides are asymptomatic [7]. However, they may acutely present with a serpiginous skin rash or gastrointestinal symptoms such as diarrhea, vomiting, and epigastric pain [7]. Patients with chronic Strongyloides infections tend to have additional respiratory symptoms such as cough, rhonchi, and dyspnea [7]. In rare instances, patients may progress to Strongyloides hyperinfection syndrome (SHS) due to increased larval migration and burden, primarily affecting the gastrointestinal and respiratory systems [8]. Strongyloides-infected patients are at increased risk of progressing to SHS in the event of an immunocompromised state [8], with the mortality rate ranging from 15% to 87% [9], and complications such as sepsis, renal failure, and respiratory failure [8].

Identifying SHS is difficult, as the diagnosis depends on the patient's clinical presentation, and the symptoms are often variable and nonspecific [8]. Also, screening a patient for Strongyloides via high sensitivity and specificity serological testing [1] is not routine practice among physicians, resulting in numerous missed cases [8]. Physicians should consider assessing immunocompromised patients with symptoms concerning Strongyloides from endemic regions earlier, to prevent the progression to SHS and decrease the risk of mortality.

## Case presentation

The patient was a 71-year-old Caucasian male residing in Charleston, West Virginia, with a past medical history of hypertension, hyperlipidemia, type II diabetes mellitus, atrial fibrillation (Afib), chronic obstructive pulmonary disease (COPD), rheumatoid arthritis, osteoporosis, class III obesity (body mass index of 43.12 kg/m^2^), restless leg syndrome, tobacco dependence, and coronary artery disease with left anterior descending artery stent placement in 2011. The patient presented to the emergency department (ED) on June 28, 2024, due to tachycardia at 176 beats per minute and dizziness while watching a video on his phone. The patient’s electrocardiogram (EKG) showed Afib with rapid ventricular response (RVR) and was hence placed on intravenous (IV) Diltiazem 10 mg drip, converting him to sinus rhythm (SR). He was discharged the same day and advised to follow up with his cardiologist.

The patient contacted emergency medical services (EMS) on June 30, 2024, with dyspnea and generalized weakness, resulting in subsequent collapse at home. Per EMS, the patient was tachypneic and hypoxic on presentation and was unresponsive to continuous positive airway pressure (CPAP). He was intubated by EMS using succinylcholine and ketamine and required additional post-intubation sedation using ketamine and rocuronium. On arrival at the ED, the patient was febrile (38.3 °C), tachycardic, and hypercarbia, with a brief period of his fever being 39.8 °C. Labs on admission were significant for elevated white blood cell (WBC) count of 20,400 with elevated absolute neutrophil (18,300) and eosinophil counts (800). Venous blood gas (VBG) showed a pH of 7.14, pCO2 80 mmHg, pO2 52 mmHg, and HCO3- 26 mmHg. A 7-French (Fr) triple-lumen catheter was placed in the right internal jugular vein, and a 5-Fr arterial line was placed in the right femoral artery. 

Sepsis protocol was initiated, and the patient was placed on IV fluids and piperacillin-tazobactam (Zosyn) 4.5 g every 8 hours (q8h) IV push (IVP). He also had elevated high sensitivity troponin (hsT) of 259 ng/L, and EKG showing SR with nonspecific ST-T-wave changes and frequent ventricular ectopy. He became increasingly hypotensive (systole in 70s mmHg) and was placed on IV norepinephrine (Levophed) 4 mg drip. The patient’s fever did not respond to oral acetaminophen 1000 mg, or IV ketorolac 15 mg. Imaging in the form of a chest X-ray (CXR), head and cervical spine computerized tomography (CT), and CT angiography (CTA) of the chest was unremarkable. A urinalysis was weakly positive for leukocyte esterase, WBCs, bacteria, and mucus. Creatinine kinase was elevated at 608 U/L, and out of concern for malignant hyperthermia, the patient was given dantrolene 360 mg IVP. The patient’s COPD was aggressively treated with an albuterol nebulizer and methylprednisolone 60 mg IVP. He also developed multiple runs of ventricular tachycardia (VT) and was cardioverted successfully to SR after three shocks at 120, 150, and 200 J. He was subsequently placed on an IV amiodarone 150 mg drip for rhythm control. Repeat labs showed an increased white blood cell count (WBC) of 38,200, a high-sensitivity troponin (hsT) level of 6,762 ng/L, hyponatremia with a sodium level of 125 mEq/L, and elevated creatinine (Cr) of 2.1 mg/dL. Arterial blood gas (ABG) demonstrated a pH of 7.21, pCO2 57 mmHg, pO2 84 mmHg, and HCO3- 22 mmHg.

Once stabilized, the patient was transferred to the intensive care unit (ICU) and was placed on a pressor regimen of IV angiotensin II 2.5 mg, Levophed 4 mg, phenylephrine 50 mg, and vasopressin 20 units (U), and a sedation regimen of IV propofol 1,000 mg, and fentanyl 2,500 mg. He was placed on an antibiotic regimen of IV Zosyn 4.5 g q8h and IV Linezolid 600 mg every 12 hours (q12h), and deep vein thrombosis (DVT) prophylaxis (PPX) using subcutaneous Heparin 5,000 U three times daily (TID). His DVT PPX was then switched to IV Heparin 3000 U PRN (if activated thromboplastin time [aPTT] <38 s), and 1,000 U PRN (if aPTT is between 38 and 49.9 s).

**Figure 1:**
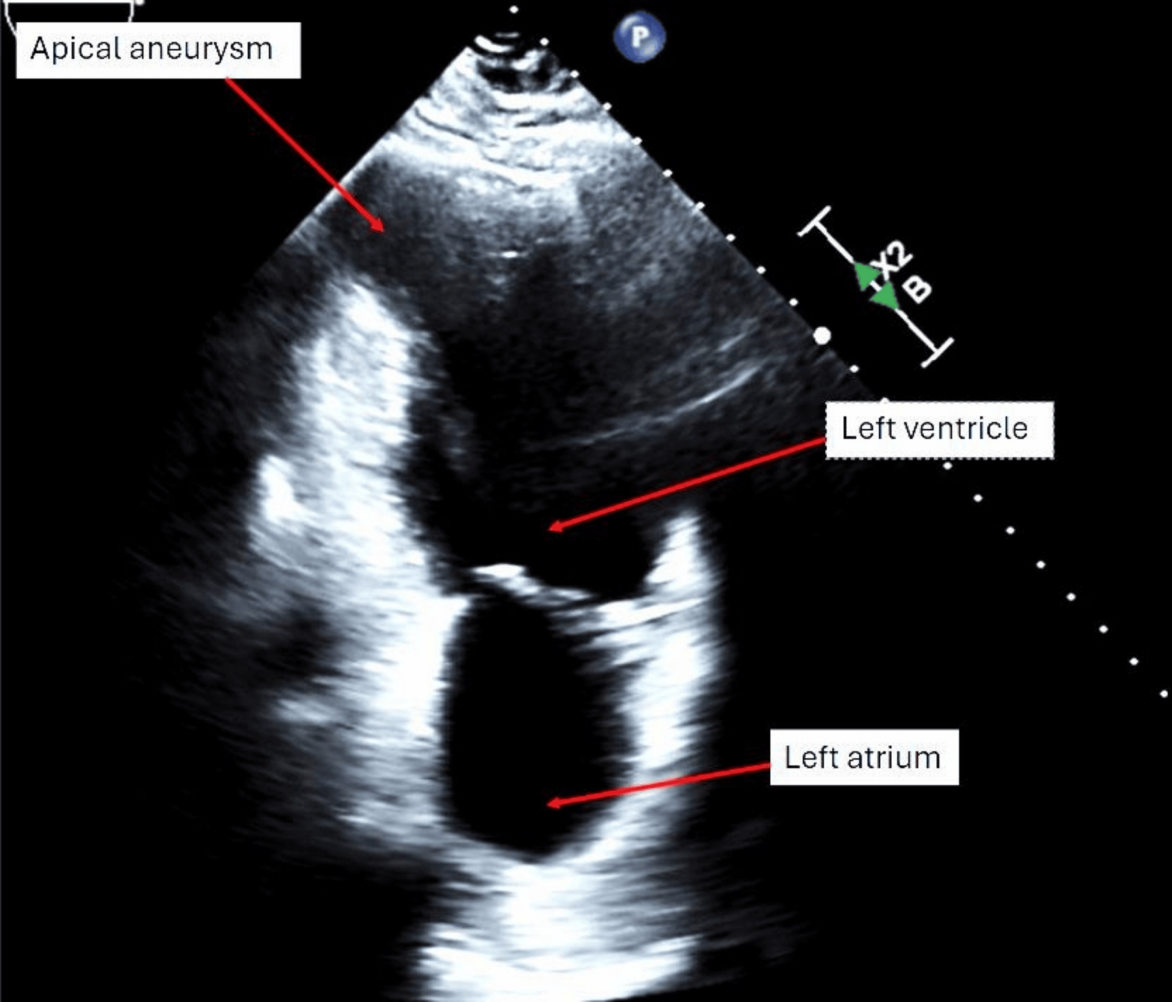
Echocardiogram from June 30 depicting an apical aneurysm.

**Figure 2:**
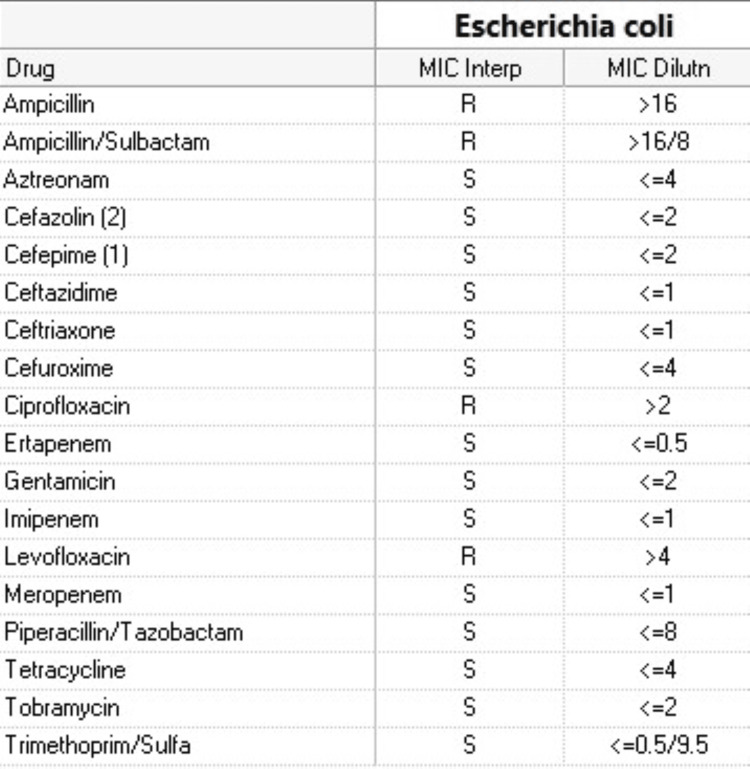
Escherichia coli antibiotic sensitivities.

A pan culture (including urine, blood, and endotracheal aspirate respiratory cultures [Cx]), immunochemistry lateral flow assay for Legionella and *Streptococcus pneumoniae* urine antigen (UA), polymerase chain reaction (PCR) for Methicillin-resistant Staphylococcus aureus (MRSA) screen, and echocardiogram were ordered. Additionally, cardiology and nephrology were consulted to manage his elevated hsT and acute kidney injury, respectively. The echocardiogram showed severe systolic dysfunction with an ejection fraction (EF) of 25%-30%, along with severe diffuse hypokinesis and an apical aneurysm (Figure 1). However, no valvulopathies or pericardial effusion were noted. The MRSA screen returned positive, and the urine and blood cultures (Cx) grew *Escherichia coli* resistant to ampicillin, ciprofloxacin, and levofloxacin (Figure 2). However, the Legionella and *S. pneumoniae* UAs and Cx were negative.

On July 1, cardiology ruled the patient was having a type II non-ST-elevation myocardial infarction (NSTEMI), and given his severe systolic dysfunction suggested performing an ischemic evaluation once the patient was more hemodynamically stable. Fick hemodynamics calculations showed cardiac output (CO) 5.9 L/minute, cardiac index (CI) 2.1 L/minute/m^2^, cardiac power output (CPO) 0.92 W, and systemic vascular resistance (SVR) 703 dynes/second/cm^2^. Cardiology recommended Swan-Ganz catheter placement for regular hemodynamic monitoring. The patient’s Levophed and vasopressin were discontinued, angiotensin II was titrated to 10 ng/kg/minute, and epinephrine was titrated to 2 mcg/minute. The patient’s Zosyn and propofol were discontinued and placed on IV Cefepime 2 g q12h, due to higher antibiotic sensitivity, while fentanyl was further titrated to 150 mcg/kg/minute. Nephrology recommended supportive therapy, including discontinuing nephrotoxic medications and monitoring Cr levels to assess the possible need for hemodialysis.

On July 3, the patient failed the CPAP trial and sedation holiday and had elevated liver enzymes with an alanine and aspartate aminotransferase (ALT/AST) of 3147/2301 U/L. Amiodarone was temporarily discontinued, and a right upper quadrant abdominal ultrasound showed hepatic changes consistent with hepatic steatosis versus intrinsic hepatocellular disease, along with multiple liver cysts and hepatomegaly. The patient was placed back on ventilator settings of pressure-regulated volume control (PRVC) with positive end-expiratory pressure (PEEP) of 5 cm H2O, a fraction of inspired oxygen (FiO2) 50%, and respiratory rate (RR) of 24 breaths/minute.

Between July 3 and July 5, the patient had no acute events and was gradually weaned off all pressures while his blood pressure remained stable. He was placed on IV dexmedetomidine (Precedex) 4 mg (titrated at 0.4 mcg/kg/hour) for sedation, while continuing fentanyl at 150 mcg/kg/minute. His ventilator settings were decreased to FiO2 40%, PEEP 5 cm H2O, RR 24 breaths/minute, and tidal volume (TV) 600 mL. He was also started on tube feeding via an 18Fr orogastric tube. He was also discontinued from cefepime and linezolid and placed on IV ceftriaxone 2 g every 12 hours. On July 5th, the patient underwent a bilateral heart catheterization (BHC) and placement of a 6Fr Swan-Ganz catheter in the left internal jugular vein. The right heart catheterization (RHC) showed a right atrial pressure of 10 mmHg, a right ventricular pressure of 65/17 mmHg, a pulmonary artery pressure of 50/24 mmHg, and a pulmonary capillary wedge pressure of 18-20 mmHg. The hemodynamic evaluation demonstrated a CO of 7.67 L/min, CI of 2.94 L/min/m², and SVR of 740 dynes·s/cm⁵. Left heart catheterization revealed a patent left main artery, mild disease of the proximal LAD, a patent mid-LAD stent, 75% stenosis of the mid-to-distal LAD, and 100% occlusion of the distal LAD. It also showed 30% ostial stenosis of the diagonal artery, a patent left circumflex artery with three patent obtuse marginal branches, a patent right coronary artery, and 75% stenosis of the proximal posterior descending artery. He was temporarily placed back on IV Levophed 4 mg after his BHC due to being hypotensive.

**Figure 3:**
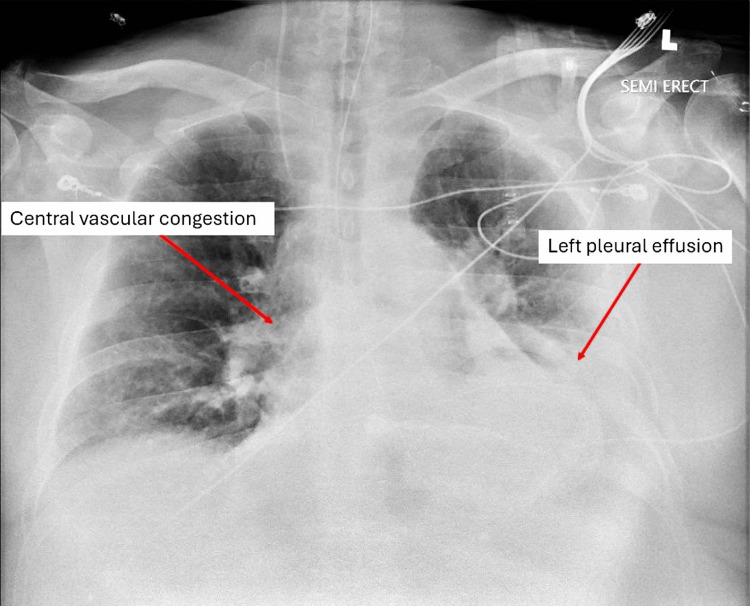
CXR from July 9 showing central vascular congestion and a left pleural effusion. CXE, chest X-ray

The Swan-Ganz catheter was subsequently removed on July 6t, and the patient’s ceftriaxone regimen was completed. On July 7, the patient was noted to be dyssynchronous on the ventilator, and his sedation regimen of fentanyl and Precedex was up-titrated. The medication regimen of Sacubitril-Valsartan (Entresto), carvedilol, and Empagliflozin (Jardiance) was discontinued due to the patient needing pressure support. Another CPAP trial failed due to the patient being agitated. Hemodynamics on July 8 demonstrated a CO of 8.4 L/minute, CI of 3.3 L/minute/m2, and SVR 428 dynes/second/cm2, suggestive of sepsis with poor cardiac reserve. Cardiology planned to perform a viability study once the patient’s condition had stabilized. Another CPAP trial failed on July 9, and the repeat pneumonia panel returned negative. CXR on July 9 demonstrated central vascular congestion, which had been present since July 1 on serial CXRs, and a left pleural effusion with associated consolidation, both of which had been stable since July 1 (Figure 3).

On July 10, the patient was placed on pressure assist-control ventilation (PACV) due to an inability to tolerate PRVC and ventilator dyssynchrony. CXR demonstrated a slightly increased moderate pleural effusion with additional consolidation. The patient was given cisatracurium (Nimbex) and aggressively diuresed with IV furosemide 40 mg BID. Despite diuresis, there was concern that the patient would require a chest tube to drain the effusion and a tracheostomy tube and percutaneous endoscopic gastrostomy (PEG) tube due to extended intubation. Due to findings of pulmonary hypertension on the RHC and severe systolic dysfunction, the patient was started on sildenafil 10 mg three times daily (TID) and Jardiance 10 mg daily. 

**Figure 4:**
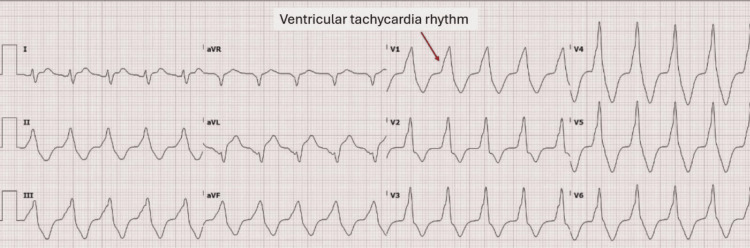
EKG from July 11 showing ventricular tachycardia. EKG, electrocardiogram

On July 11, the patient had a cardiac arrest secondary to VT for three minutes, requiring one round of cardiopulmonary resuscitation, and IV epinephrine 1 mg, which converted the patient to a supraventricular tachycardia, which spontaneously converted to SR (Figure 4). The patient’s Levophed was further up-titrated to maintain a mean arterial pressure of 65 mmHg. The patient’s fentanyl was increased back to 150 mcg/kg/minute and was given a bolus of amiodarone 150 mg IVP and lidocaine 100 mg IVP with subsequent IV lidocaine 2 g drip. The patient’s Precedex and sildenafil were also discontinued. The echocardiogram showed an EF of 60% without any wall motion abnormalities. Cardiology recommended no revascularization, and electrophysiology decreased IV lidocaine to 1 g, with recommendations for deep sedation in the event of recurrent VT. On July 13, the patient was in accelerated idioventricular rhythm and had episodes of ventilator dyssynchrony, requiring Nimbex administration.

**Figure 5:**
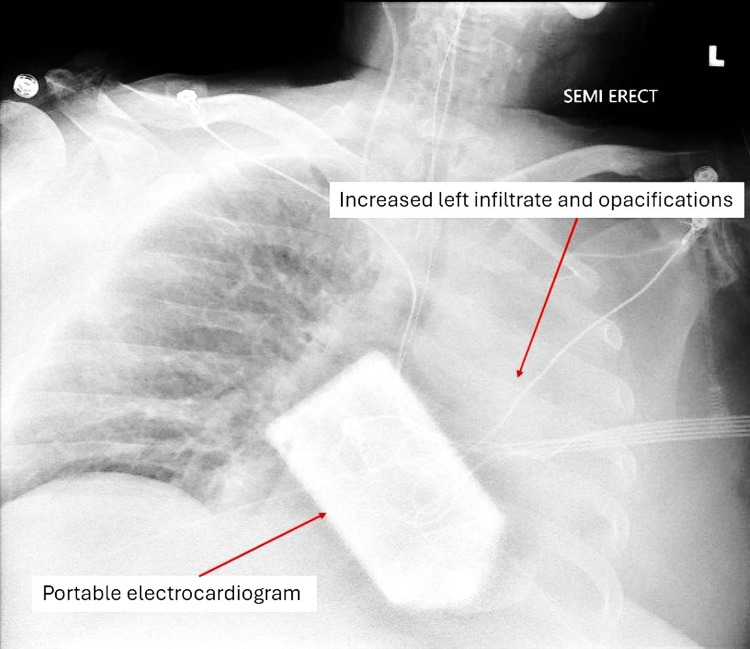
CXR from July 14 showing worsening left lung opacifications and infiltrate.

CXR on July 14 showed increased opacification and infiltration of the left lung (Figure 5). The patient underwent bronchoscopy, which revealed significant mucus plugging in the left lower lobe. Respiratory Cx obtained from sputum during bronchoscopy grew non-drug-resistant *E. coli*, which was treated with Zosyn 4.5 g q8h. Mucus plugging continued to show on the CXR on July 18, and a repeat bronchoscopy with bronchoalveolar lavage (BAL) of the left lung was performed. Between July 18 and July 21, the patient’s respiratory status remained poor, with a FiO2 of 65% and PEEP ranging from 10 to 12 cm H2O. On July 21, the patient’s WBC increased from 28,500 to 54,300. A blood culture and respiratory culture of tracheal aspirate from bronchoscopy were obtained. He also underwent a tracheostomy tube and PEG tube placement on July 23. Respiratory culture returned positive for *Strongyloides stercoralis*, and the patient was started on IV ivermectin 200 mcg/kg for 14 days. He was also transitioned to IV ceftriaxone 2 g for 14 days for his *E. coli* pneumonia. The infectious disease team diagnosed the patient with SHS.

**Figure 6:**
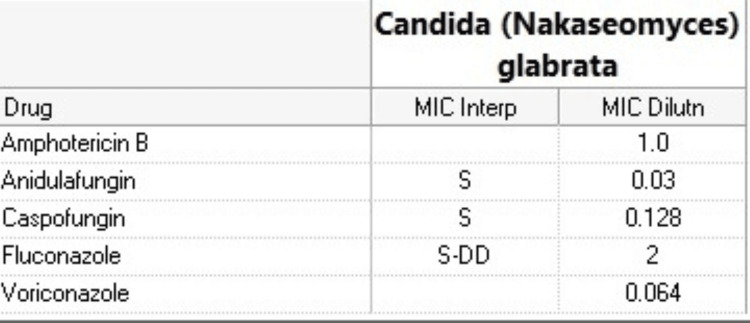
Candida glabrata antifungal sensitivities.

The patient underwent repeat bronchoscopies with BAL on July 26 and 27 due to recurrent mucus plugging affecting the left lung. Due to recurrent febrile episodes, paired blood cultures were drawn, showing growth of *Candida glabrata*. The patient was given an IV anidulafungin 200 mg loading dose (Figure 6). On July 28, the patient’s oxygen was desaturated to 70s% despite being on maximum ventilator settings with a FiO2 of 100%, and PEEP of 14 cmH2O. He also had a lactic acid of 10.8mmol/L and an arterial pH of 7.18. After discussions with the patient’s surrogate, the daughter, regarding the patient’s worsening respiratory status, multiple infections, and increased pressure-demand, the patient was switched to do not resuscitate status, with continued intubation. The patient passed away on July 29 at 00:55.

## Discussion

This case is a perfect example of the prevalence of Strongyloides in an immunocompromised patient from the Appalachian region. The prolonged ICU stay, secondary to multiple episodes of VT, acute on chronic respiratory failure, severe sepsis from E. coli bacteremia, pneumonia, and urinary tract infections, and congestive heart failure with reduced ejection fraction, placed the patient at high risk of developing SHS from a likely preexisting undetected Strongyloides infection. 

Given the multitude of medical problems that the patient was experiencing while in the ICU, it is easy for physicians to overlook a possible underlying Strongyloides infection. This raises the question: Should immunocompromised patients from the Appalachian region who present with appropriate symptoms be screened for Strongyloides? Prior studies have shown that acute respiratory failure, though nonspecific, is a common symptom of patients with SHS [10,11]. Patients with an underlying Strongyloides infection tend to have symptoms that mimic COPD exacerbation [12], which can progress to respiratory failure. The patient’s inability to be weaned from the ventilator to CPAP, increased oxygenation demand, and recurrent mucus plugging noted on bronchoscopy suggest an underlying infectious state that is preventing improvement in respiratory status. Given the patient’s clinical status, earlier screening for Strongyloides may have provided a more complete initial workup.

Similar to this case, other previously documented cases have acquired gram-negative sepsis in the setting of SHS [10,12]. The rationale for the additional findings of gram-negative sepsis is due to the Strongyloides larvae facilitating the invasion of gram-negative bacteria from the bowel mucosa into the circulation [12]. A study conducted in Cochabamba, Bolivia, involving 149 end-stage renal disease patients, noted that 57.3% of the patients developed septic shock attributed to larvae penetration of the bowel mucosa, facilitating the translocation of enteric bacteria into circulation [13]. Given the high frequency of gram-negative sepsis in the setting of SHS as noted in prior studies, physicians should consider testing patients with appropriate symptoms and similar findings for the Strongyloides parasite. On re-evaluation of this case, the findings of E. coli bacteremia and acute respiratory failure with accompanying E. coli pneumonia should prompt earlier screening for the Strongyloides parasite.

It is reassuring for physicians that there are clinical presentations that can be suggestive of an underlying Strongyloides infection. However, the question remains if early detection would decrease mortality or extend a patient’s duration of life. Prior studies have suggested that early detection of Strongyloides can prevent mortality by treating subclinical infections [6]. Other papers have also suggested prophylactic treatment with albendazole, ivermectin, and thiabendazole, to prevent infection of high-risk immunocompromised patients like transplant patients [6,14]. A prior study assessing the risk of Strongyloides infection in 138 renal transplant patients receiving prophylactic therapy showed a delayed onset of infection in patients receiving prophylaxis with albendazole, ivermectin, and thiabendazole, compared to the control group (182 days vs. 87.5 days) [14]. The paper also showed that the prophylactic treatment group had a lower mortality rate of 14%, compared to a mortality rate of 50% in the control group [14].

Based on these prior studies, there is evidence to suggest that early detection of Strongyloides, or the use of prophylactic treatment, could decrease the mortality rate. One of the limitations of the available evidence is that there are insufficient studies assessing this medical management in ICU patients. Regarding the discussed case, the patient's multimorbidities and acute conditions increased his risk of mortality, and it is debatable whether early detection of Strongyloides would have prolonged his life expectancy.

## Conclusions

In summary, physicians managing symptomatic immunocompromised individuals in endemic regions should consider early screening for Strongyloides via serological testing and reduce the risk of SHS and subsequent mortality. Medical professionals should be mindful of the limited studies on Strongyloides, which can be attributed to a lack of parasite screening. Future studies should assess the effectiveness of screening techniques or prophylactic measures on ICU patients or other immunocompromised groups to decrease Strongyloides-related deaths.

## References

[1] Yang R, Xu M, Zhang L, Liao Y, Liu Y, Deng X, Wang L (2024). Human Strongyloides stercoralis infection. J Microbiol Immunol Infect.

[2] Russell ES, Gray EB, Marshall RE (2014). Prevalence of Strongyloides stercoralis antibodies among a rural Appalachian population--Kentucky, 2013. Am J Trop Med Hyg.

[3] Nutman TB (2017). Human infection with Strongyloides stercoralis and other related Strongyloides species. Parasitology.

[4] Buonfrate D, Bisanzio D, Giorli G (2020). The global prevalence of Strongyloides stercoralis infection. Pathogens.

[5] Yates J (2021). Parasitic infections: do not neglect strongyloidiasis. Am Fam Phys.

[6] Yeh MY, Aggarwal S, Carrig M (2023). Strongyloides stercoralis infection in humans: a narrative review of the most neglected parasitic disease. Cureus.

[7] Mora Carpio AL, Meseeha M (2024). Strongyloidiasis. https://www.ncbi.nlm.nih.gov/books/NBK436024/.

[8] Kassalik M, Mönkemüller K (2011). Strongyloides stercoralis hyperinfection syndrome and disseminated disease. Gastroenterol Hepatol (N Y).

[9] Marcos LA, Terashima A, Dupont HL, Gotuzzo E (2008). Strongyloides hyperinfection syndrome: an emerging global infectious disease. Trans R Soc Trop Med Hyg.

[10] Tsai M, Wu T, Tsai K (2011). Acute respiratory distress syndrome complicating Strongyloides stercoralis hyperinfection. Int J Gerontol.

[11] Nnaoma C, Chika-Nwosuh O, Engell C (2019). The worm that clogs the lungs: Strongyloides hyper-infection leading to fatal acute respiratory distress syndrome (ARDS). Am J Case Rep.

[12] Newberry AM, Williams DN, Stauffer WM, Boulware DR, Hendel-Paterson BR, Walker PF (2005). Strongyloides hyperinfection presenting as acute respiratory failure and gram-negative sepsis. Chest.

[13] Tebib N, Tebib N, Paredes M (2023). Prevalence and risk factors of Strongyloides stercoralis in haemodialysis in Cochabamba, Bolivia: a cross-sectional study. BMC Nephrol.

[14] Miglioli-Galvão L, Pestana JO, Santoro-Lopes G (2020). Severe Strongyloides stercoralis infection in kidney transplant recipients: a multicenter case-control study. PLoS Negl Trop Dis.

